# Management-driven evolution in a domesticated ecosystem

**DOI:** 10.1098/rsbl.2013.1082

**Published:** 2014-02

**Authors:** Vigdis Vandvik, Joachim P. Töpper, Zoë Cook, Matthew I. Daws, Einar Heegaard, Inger E. Måren, Liv Guri Velle

**Affiliations:** 1 Department of Biology, University of Bergen, Bergen, Norway; 2 Department of Geography, University of Bergen, Bergen, Norway; 3 Royal Botanic Gardens Kew, Wakehurst Place, West Sussex, UK; 4 Norwegian Forest and Landscape Institute, Fana, Norway; 5 Norwegian Institute for Agricultural and Environmental Research, Fureneset, Norway; 6 Faculty of Engineering and Science, Sogn og Fjordane University College, Sogndal, Norway

**Keywords:** smoke-induced germination, fire, coastal heathland, germination cues, cultural landscape

## Abstract

Millennia of human land-use have resulted in the widespread occurrence of what have been coined ‘domesticated ecosystems’. The anthropogenic imprints on diversity, composition, structure and functioning of such systems are well documented. However, evolutionary consequences of human activities in these ecosystems are enigmatic. *Calluna vulgaris* (L.) is a keystone species of coastal heathlands in northwest Europe, an ancient semi-natural landscape of considerable conservation interest. Like many species from naturally fire-prone ecosystems, *Calluna* shows smoke-adapted germination, but it is unclear whether this trait arose prior to the development of these semi-natural landscapes or is an evolutionary response to the anthropogenic fire regime. We show that smoke-induced germination in *Calluna* is found in populations from traditionally burnt coastal heathlands but is lacking in naturally occurring populations from other habitats with infrequent natural fires. Our study thus demonstrates evolutionary imprints of human land-use in semi-natural ecosystems. Evolutionary consequences of historic anthropogenic impacts on wildlife have been understudied, but understanding these consequences is necessary for informed conservation and ecosystem management.

## 1. Introduction

Fire is known to stimulate germination in many species of naturally fire-prone ecosystems worldwide [1,2]. Different smoke-derived chemical substances, notably karrikinolide and glyceronitrile [3,4], have been shown to play key ecophysiological roles in smoke-stimulated germination. The repeated appearance of the trait in many different families, lineages and regions [1,5–7] suggests a strong capacity for evolutionary responses to fire in plants [2], and hence potential for convergent evolution. Humans have used burning as a management tool for millennia [8,9], and fire has strong impacts on the structure and functioning of the resulting semi-natural ecosystems [8,10], suggesting that culturally fire-prone habitats may be good candidate systems for studying evolutionary responses to human management regimes.

The coastal heathlands of northwest Europe constitute an anthropogenic landscape that has been continuously managed by traditional burning and grazing regimes for up to 6000 years [9–11]. Major expansion occurred from *ca* 5000 BP (before present) in Jutland, Denmark [12], and at 3300–1000 BP in western Norway [11,13]; and although studies are scarce in the north, there is evidence of anthropogenic coastal heathland 4700–3300 BP in central Norway [14], and 3800–1800 BP in northern Norway [15]. Burning cycles of 10–20 years are traditional throughout the coastal heathland region [10,16]. Smoke-stimulated germination responses in heathland species, e.g. *Calluna vulgaris* [17,18], have ecological consequences: for example, germination rate and final percentages increase in smoke-exposed seedbanks [17,19,20]. As 6000 years of human influence affords scope for evolutionary change, we ask: is this a trait that *Calluna* brought into the heathlands or has it evolved there?

**Figure 1.**
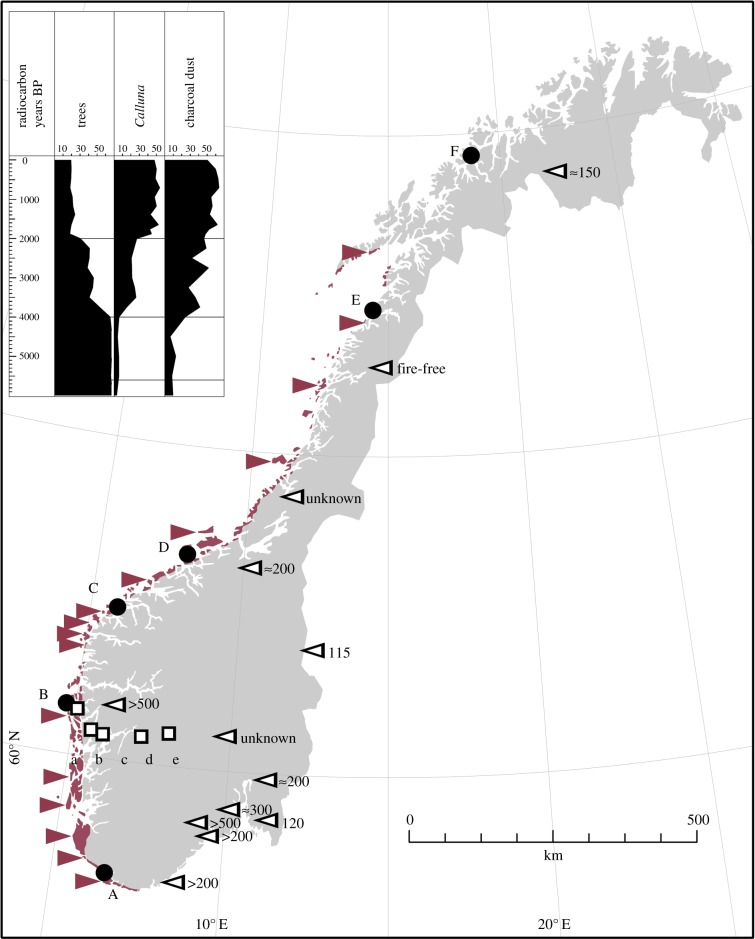
Fire frequencies, study sites and distribution of anthropogenic coastal heathlands (purple shade) in Norway. Purple arrows indicate sites with frequent fires documented back to the Late Bronze or Iron Age (selected from 70 palaeoecological records [9,11,13–15]). White arrows indicate boreal heaths or forests with low-frequency natural fire regimes, with years since last fire given next to each arrow (from [22–25]). Black circles and white squares indicate seed-sampling sites along the latitudinal and elevational gradient, respectively. Inset shows a microfossil record from site B over the past 6000 years (reprinted with permission from [17]). See the electronic supplementary material, table S1 for site information. (Online version in colour.)

*Calluna* also has a wide distribution in natural habitats—pine forests, boreal heaths and alpine areas [21]—that have not been exposed to high-frequency anthropogenic fire regimes. Natural fire frequencies in these boreal habitats vary considerably in space and time but are much lower than those of the managed coastal heathlands [22–25]. To evaluate evolutionary impacts of the anthropogenic fire regimes, we assessed germination responses to smoke of *Calluna* seeds sampled along two geographical gradients with different fire histories: (I) a latitudinal gradient within anthropogenic heathlands along the coast of Norway and (II) an elevational gradient away from the anthropogenic coastal heathlands into boreal forests and heaths (figure 1). The gradients cover comparable climatic conditions, but whereas burning has been a common practice along the coast it has not in the boreal forests and heaths (figure 1; electronic supplementary material, table S1).

## 2. Material and methods

Eleven *Calluna* populations were studied (figure 1; electronic supplementary material, table S1). Data on fire frequencies were obtained from more than 70 fossil charcoal records from anthropogenic coastal *Calluna* heathlands (reviewed in [9,11,13–15]) and 12 records from boreal-zone *Calluna* habitats [22–25]. From each study population, infructescences from 15 *Calluna* plants were harvested, dried at 20°C for 2 days and stored for five months at 15% relative humidity and 15°C. These seeds were germinated with and without the addition of smoke water (standard *Themeda* solution; diluted 1 : 500 000 based on a dose-response screening experiment reported in the electronic supplementary material, table S2) [5]. For each maternal plant and treatment, three replicate Petri dishes of 22 seeds sown on agar were incubated at 20°C with a diurnal cycle of 16 L : 8 D; these conditions are known to yield maximum germination rates and percentages in *Calluna* [18,21,26]. Germination (radicle more than or equal to 0.5 mm) was scored for 60 days. We used a generalized linear mixed model (GLMM) solved by an integrated nested Laplace approximation [27] assuming a binomial distribution. Effects of explanatory variables—time, treatment and geography—on germination probabilities were assessed through posterior distributions using a three-way interaction model with random contributions by populations, maternal plants, replications and a term for residual overdispersion and autocorrelation. All analyses were done in R v. 2.15.2 [28].

## 3. Results

**Figure 2.**
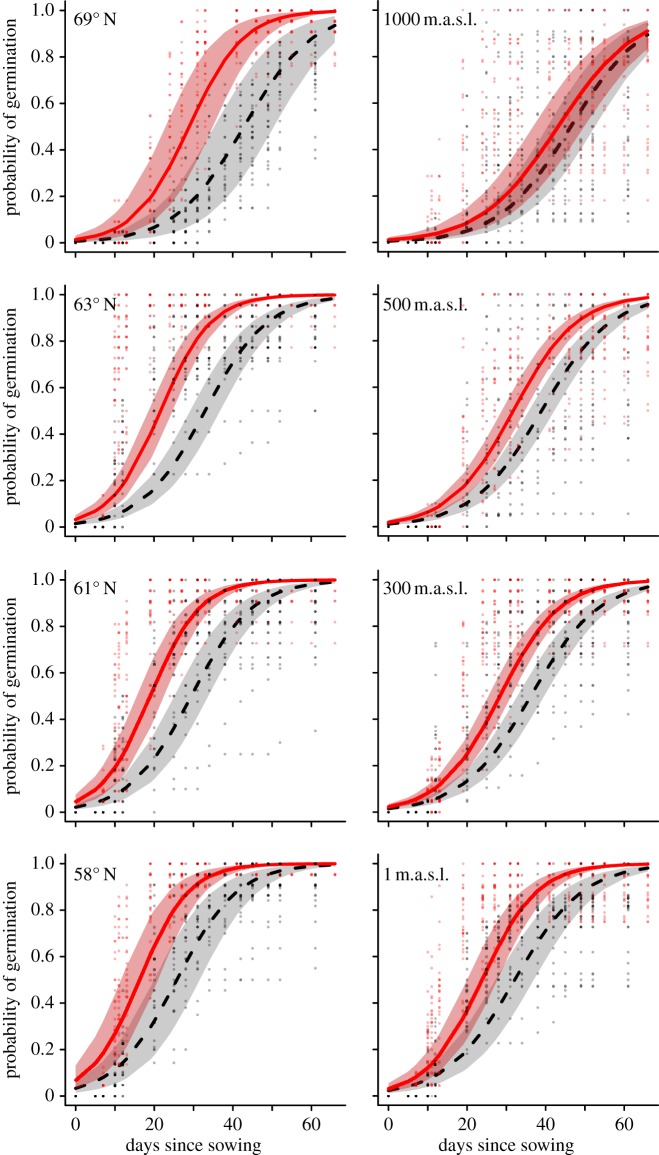
*Calluna vulgaris* germination probabilities over time in response to smoke treatment along the latitudinal and elevational gradients. Lines give model predictions (posterior distributions from GLMM) and shaded areas delimit 2.5–97.5 percentile credibility of smoke-treated (red) and control (black) samples. m.a.s.l., metres above sea level.

Fire frequencies differ sharply, by up to three orders of magnitude, between the anthropogenic coastal heathlands and other *Calluna* habitats. Outside the coastal heathland region, several sites are fire-free with median time since fire more than 200 years (figure 1). Smoke treatment increases both germination rates and final percentages in all *Calluna* populations from the latitudinal gradient (anthropogenic heath), and there are no significant interactions between smoke treatment and geography, indicating that the effect is constant along the entire gradient (table 1 and figure 2). The effect translates into an advancement of germination by 10–14 days (table 2) or a reduction in mean time to germination by 32–37%. The positive smoke-treatment effect is also detected along the elevational gradient, but here the smoke-treatment effect diminishes away from the coastal heathlands, as indicated by a negative three-way interaction with geography (table 1). At the coast, the model predicts an 8-day or 25% reduction in mean time to germination, which matches the prediction from the latitudinal gradient model, but at the boreal (natural) heath end of the gradient the difference is only 3 days and no longer statistically significant as indicated by the overlapping confidence intervals of smoke-treated seeds and controls (table 2 and figure 2). Climate effects are consistent and comparable across the two gradients: germination rates decrease towards the colder northern and mountain regions, with similar parameter estimates and predictions (tables 1 and 2).

**Table 1.** GLMM fixed effects for *C. vulgaris* seed germination over time in response to smoke treatment along two geographical gradients.

	latitudinal gradient		elevational gradient	
	estimate	s.d.	estimate	s.d.
intercept	4.999	3.832	−3.786	0.316
time	0.205	0.026	0.118	0.004
geography^a^	−1.454	0.606	−1.399	0.552
geography × time	−0.012	0.004	−0.006	0.007
smoke	0.771	0.207	0.335	0.364
smoke × time	0.031	0.003	0.027	0.005
smoke × geography			0.362	0.655
smoke × geography × time			−0.034	0.010

^a^Geographical effects are given per 1000 m.a.s.l. in the elevational gradient model, and per 10° N in the latitudinal gradient model. s.d., standard deviation.

**Table 2.** Germination rate of *C. vulgaris* seeds at 20°C expressed as mean time to 50% germination according to the models of smoke-treatment effects along the two geographical gradients (table 1). Elevation is given in m.a.s.l.

	smoke treatment	
	no	yes
latitude		
69° N	42	28
64° N	34	23
59° N	27	17
elevation		
1000 m	46	43
500 m	39	32
0 m	32	24

## 4. Discussion

Smoke-induced germination is known from *Calluna* populations in anthropogenic coastal heathlands [17,18], where it increases recruitment from seedbanks in newly burnt heath [19,20]. We document that the trait is not universally present in *Calluna*; instead, it is lacking in the species' range outside the culturally fire-prone coastal heathlands. This can be linked to fire frequencies, which are markedly higher in anthropogenic than in natural *Calluna* habitats. This suggests that the smoke response has evolved in response to the anthropogenic high-frequency fire regime: *Calluna* occurs widely in heaths, bogs, forests and alpine areas throughout Europe [21] that lack the recurrent burning characteristic of the anthropogenic heathlands. The difference in fire history inside and outside the coastal heathlands is ancient; these landscapes were cleared in the Neolithic, with a period of expansion in the Bronze Age [9–11], providing time for evolutionary differentiation. Research on smoke-responses in naturally fire-prone ecosystems documents that the trait is phylogenetically and geographically widespread and is found in both broad-ranged and endemic plant species [5–7]. This suggests that smoke-induced germination is an evolutionary convergence [2]. The hypothesis of convergent evolution has gained support from studies demonstrating that smoke-responses in different phylogenetic lineages can be triggered by the same few chemical substances, universally present in plant-derived smoke [3,4].

The use of the two contrasting geographical gradients enables us to isolate the land-use difference and avoid confounding climate effects (figure 1; electronic supplementary material, appendix S1). The similar germination responses along the climatic gradients show that the study design was successful in isolating the heathland-burning effect. Indeed, a model testing *only* climate found no significant differences in germination responses to temperature along the two gradients (not shown).

In many of the classic studies of evolutionary consequences of human activities [29], the management actions interfere directly with age-specific survival rates. By contrast, evolutionary effects on non-target species are more enigmatic. In our study system, *Calluna* populations are not harvested but subjected to a management regime that affects their life cycle. Rather than affecting fecundity or growth, this management regime has effects on germination regulation, which in turn affects recruitment success [17,20]. Our findings have implications for the biodiversity and conservation value of coastal heathlands and domesticated ecosystems in general. If these harbour distinct ecotypes adapted to anthropogenic impacts [30], biodiversity will be at risk if semi-natural habitats and associated land-use regimes disappear.

## Data accessibility

Data deposited in the Dryad repository under the DOI: http://doi.org/10.5061/dryad.191d5.
